# Supplementary material for: Salt Tolerance Diversity in Citrus Rootstocks Agrees with Genotypic Diversity at the LCl-6 Quantitative Trait Locus
Source: Genes (Basel). 2025 May 30;16(6):683. doi: 10.3390/genes16060683 (PMC12191971; doi:10.3390/genes16060683)
Supplement: Supplementary file 1 [file genes-16-00683-s001.zip › genes-3677221-supplementary.pdf]

Supplementary Materials of the article "Salt tolerance diversity in citrus rootstocks agrees with genotypic diversity at the *LCl-6* quantitative trait locus" by Maria J. Asins, V. Raga, M. Romero-Aranda, Emilio Jaime-Fernández, Emilio A. Carbonell and Andres Belver

Figure S1. Pictures of all plants per accession and treatment (C, control; S, salinity) ordered by species.

Tr-537 trifoliate orange Flying Dragon

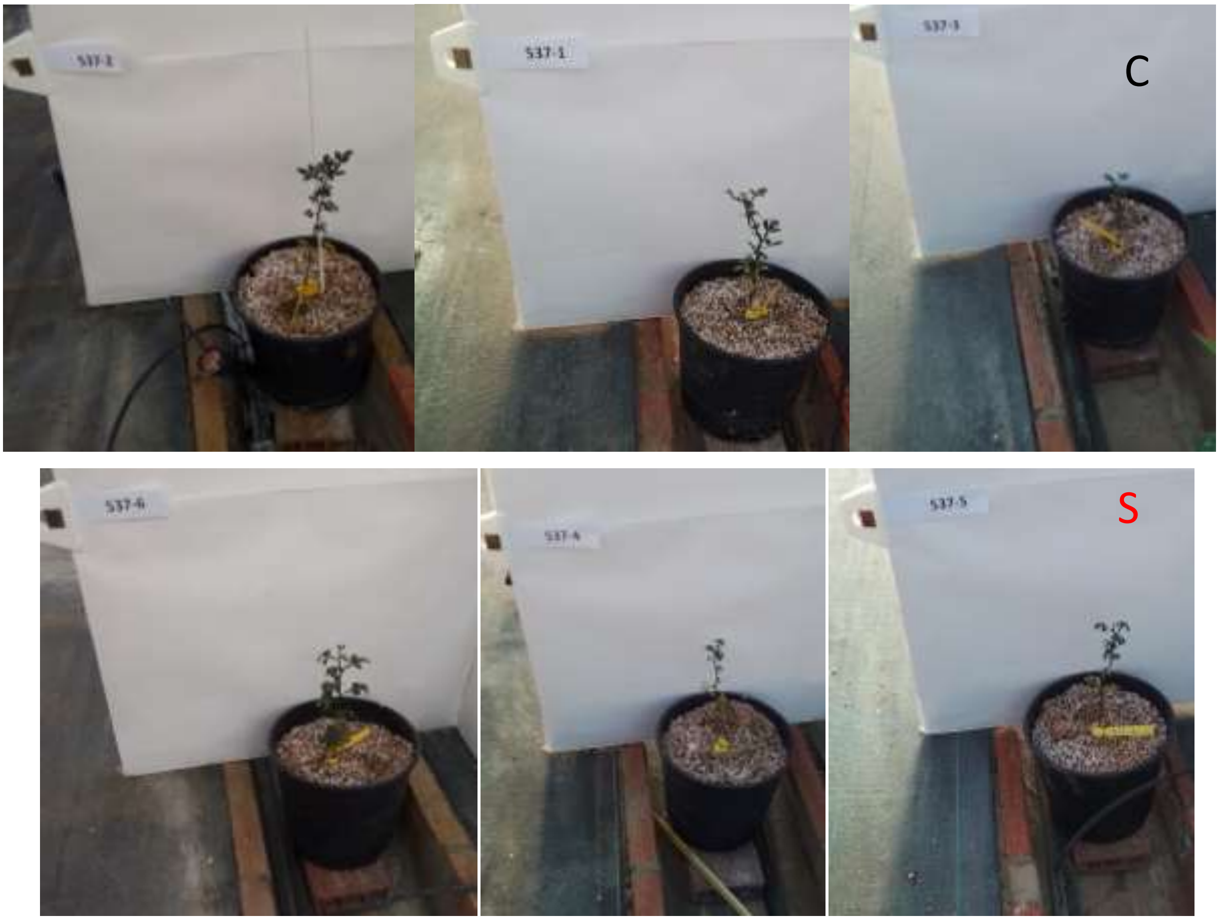

Tr-236 trifoliate orange Rich

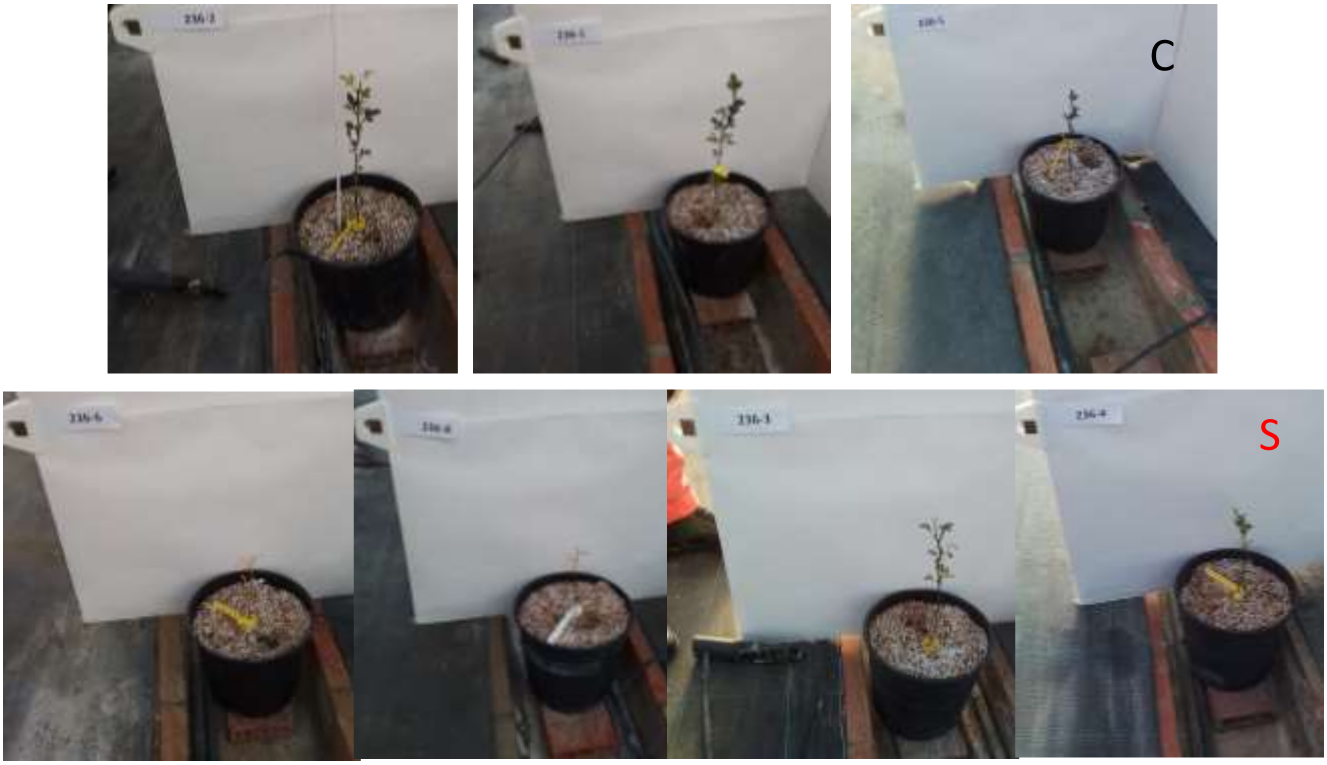

Tr-376 trifoliate orange Benecke

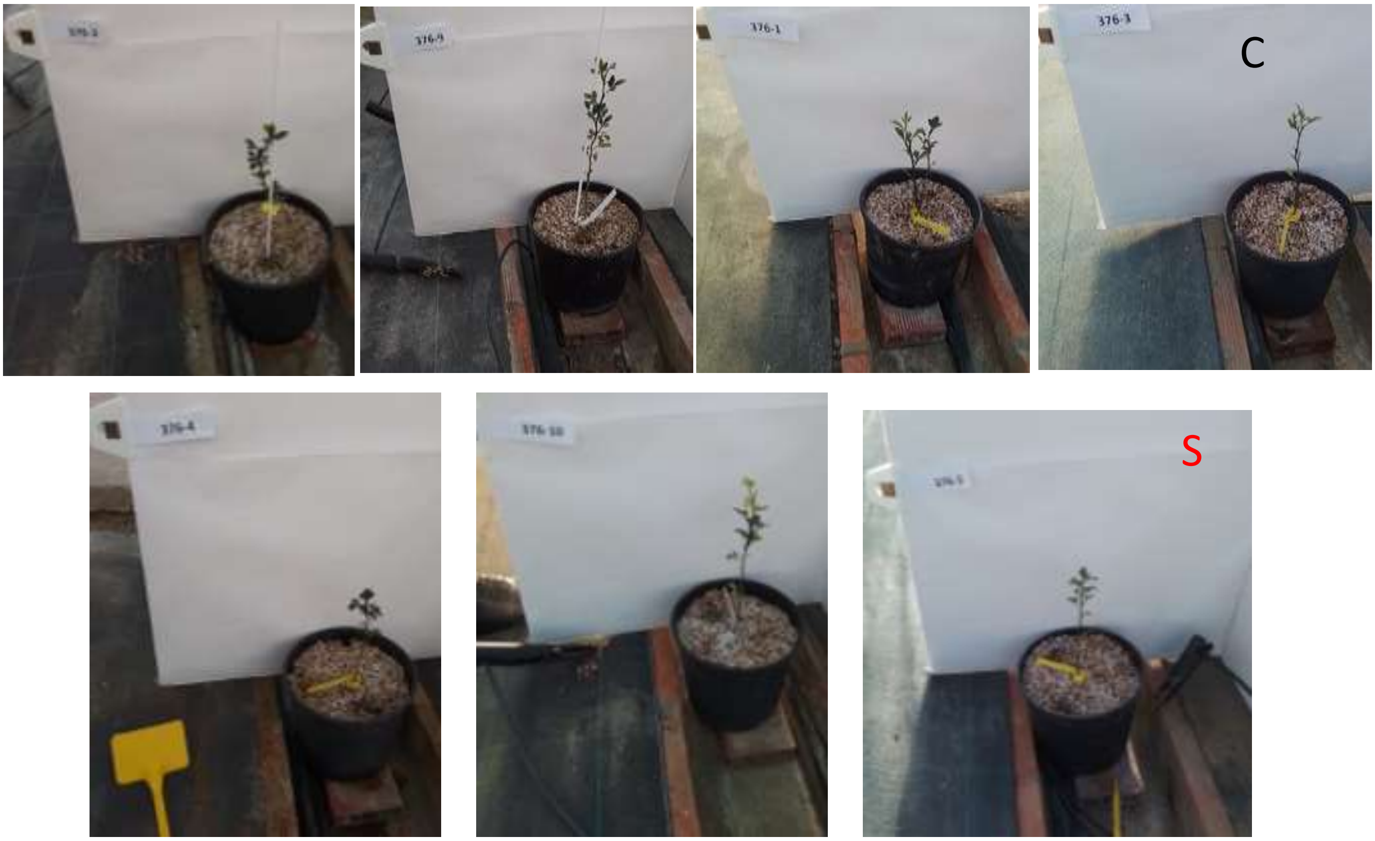

Tr-374 trifoliate orange Pomeroy

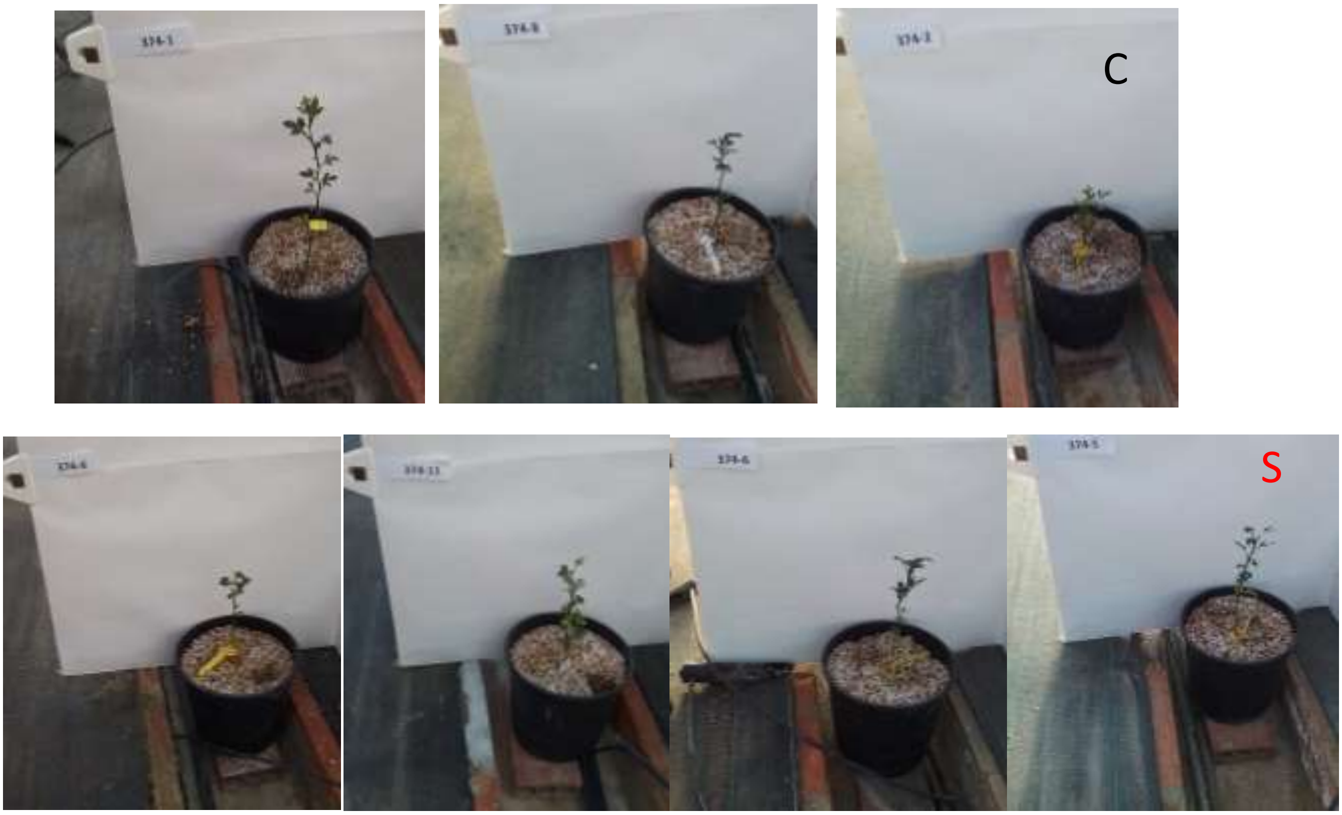

Au-130 sour orange Afin-Verna3

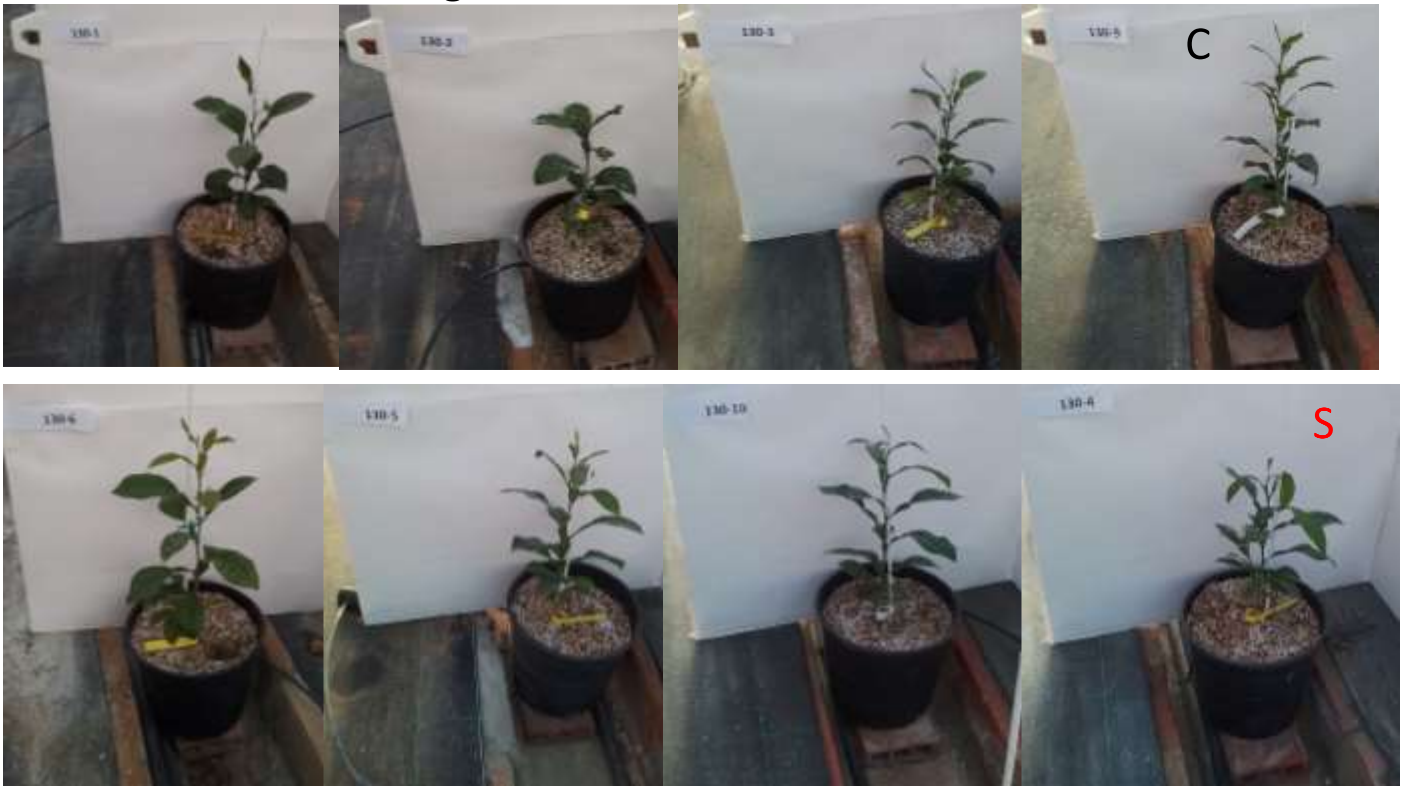

Au-141 sour orange Clementina (control)

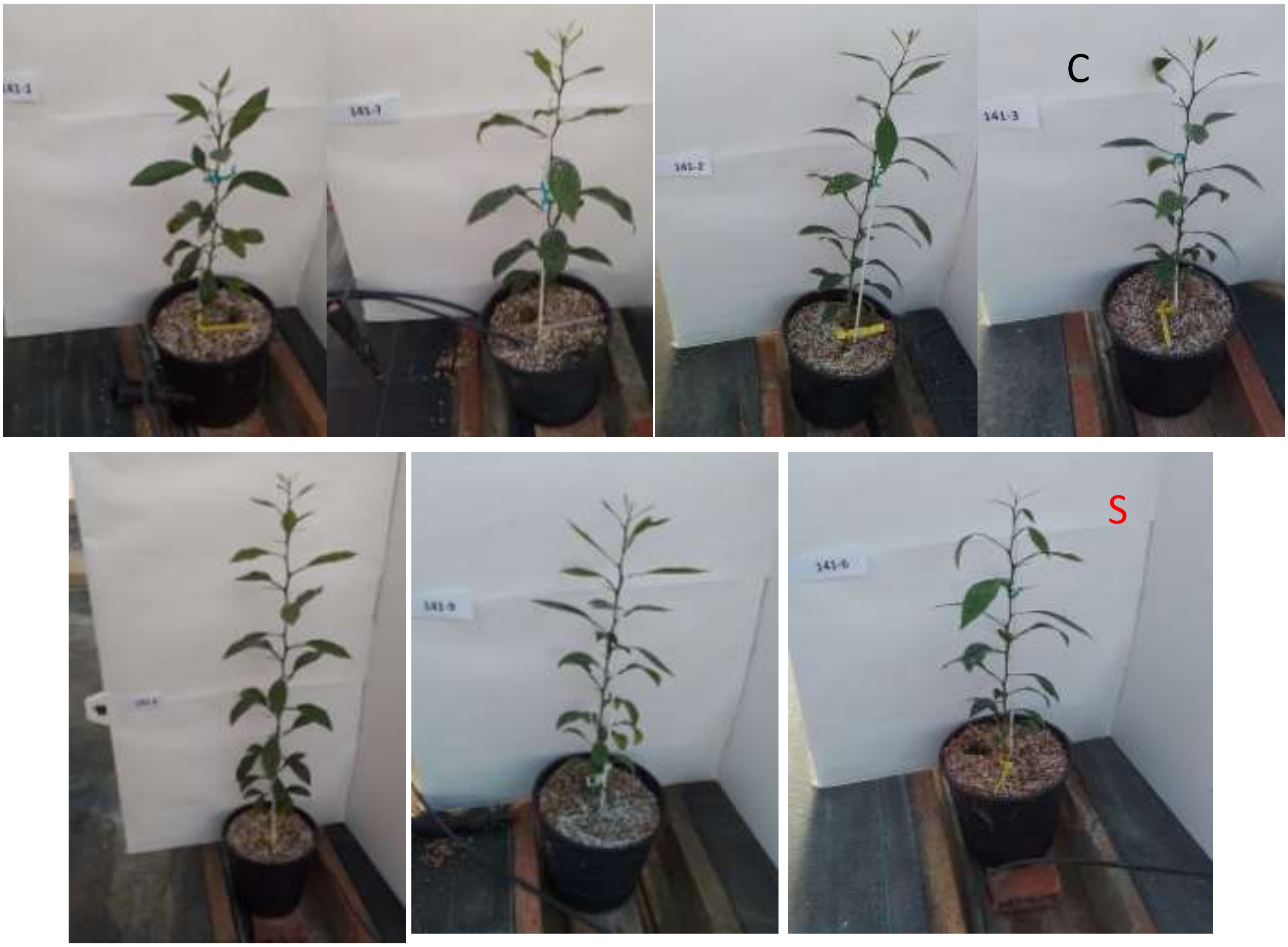

Au-183 sour orange Guo Kuo Cheng

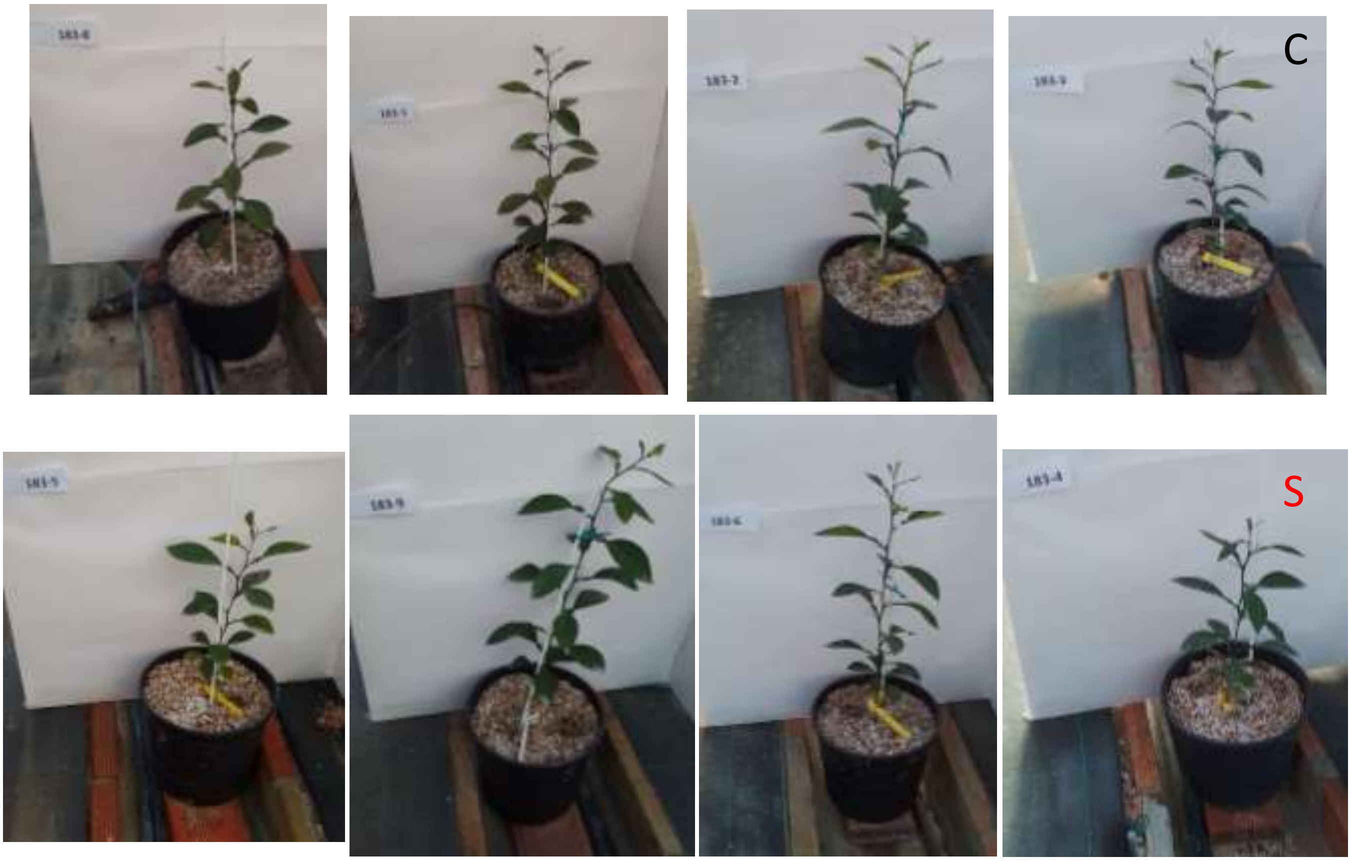

Si-011 sweet orange Pineaple

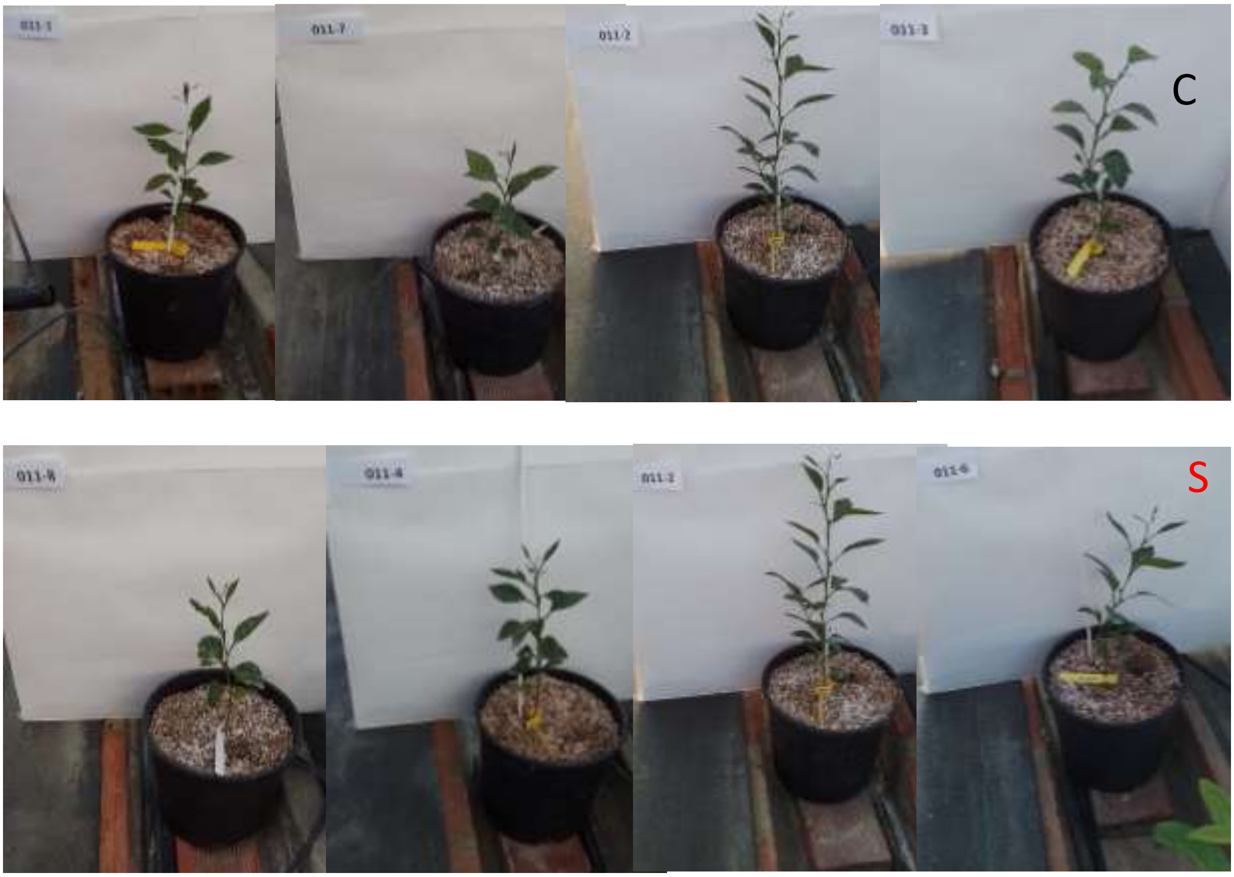

Su-239 Sunki mandarin

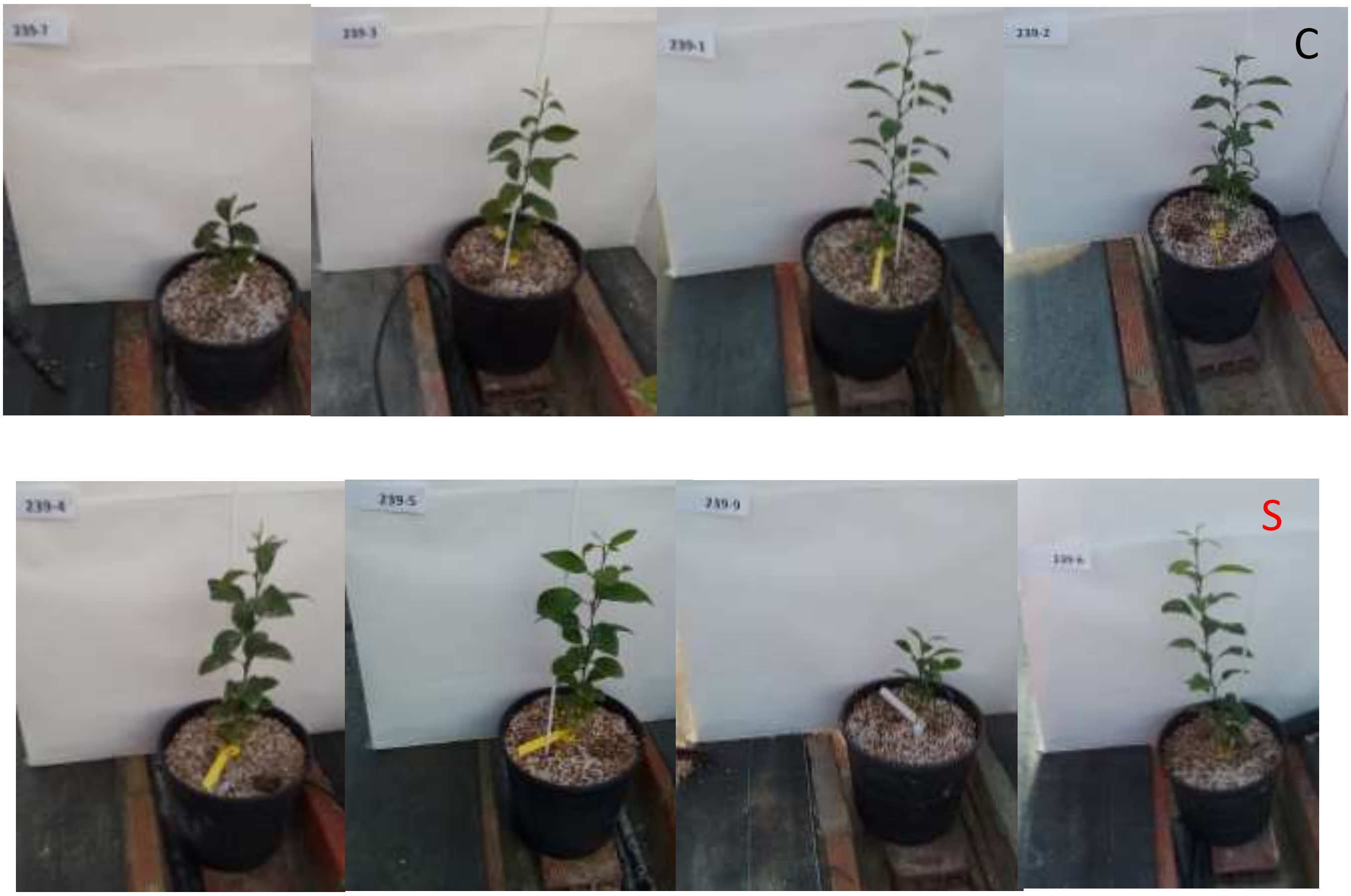

Re-385 Cleopatra mandarin

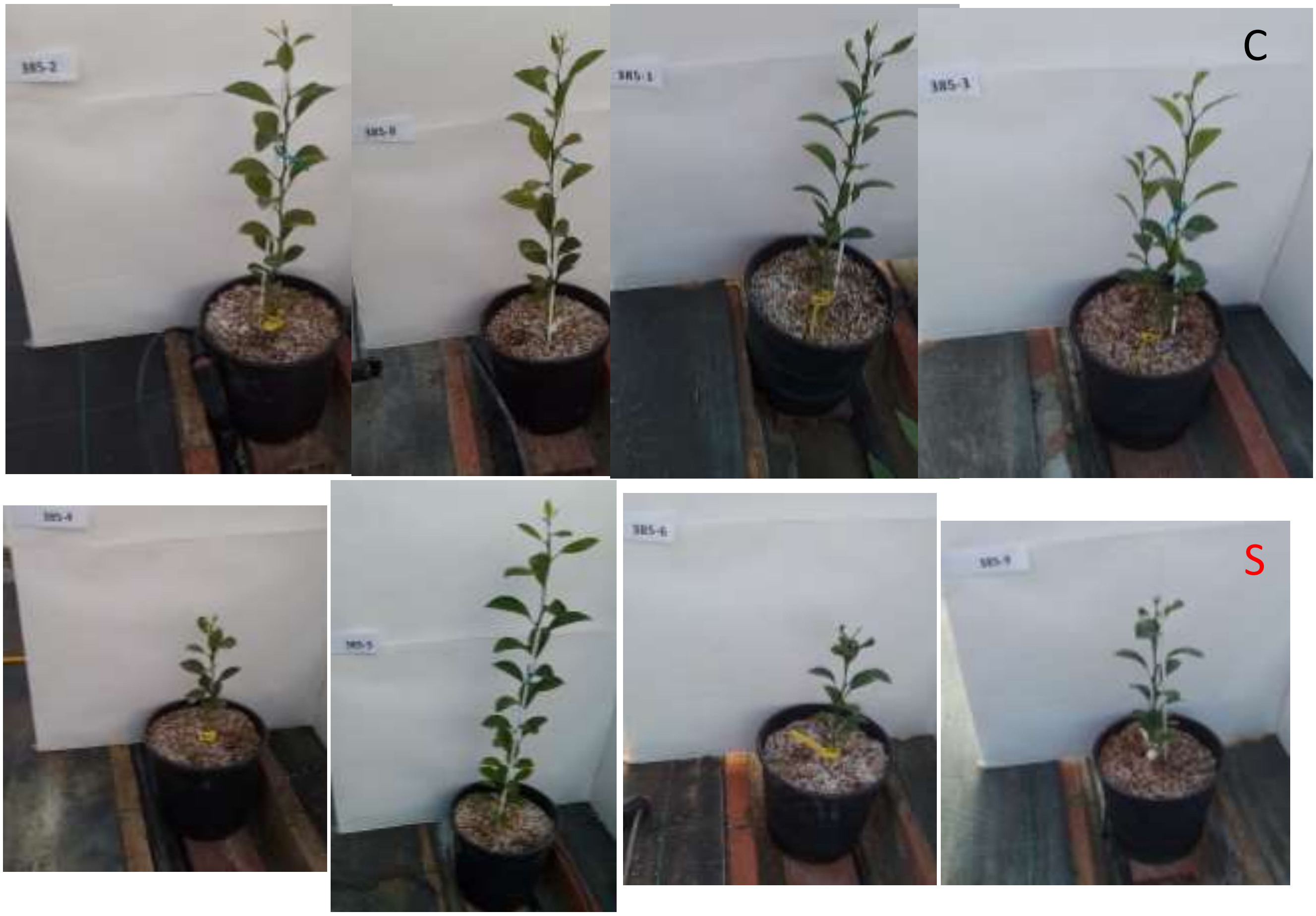

Ma-288 Alemow

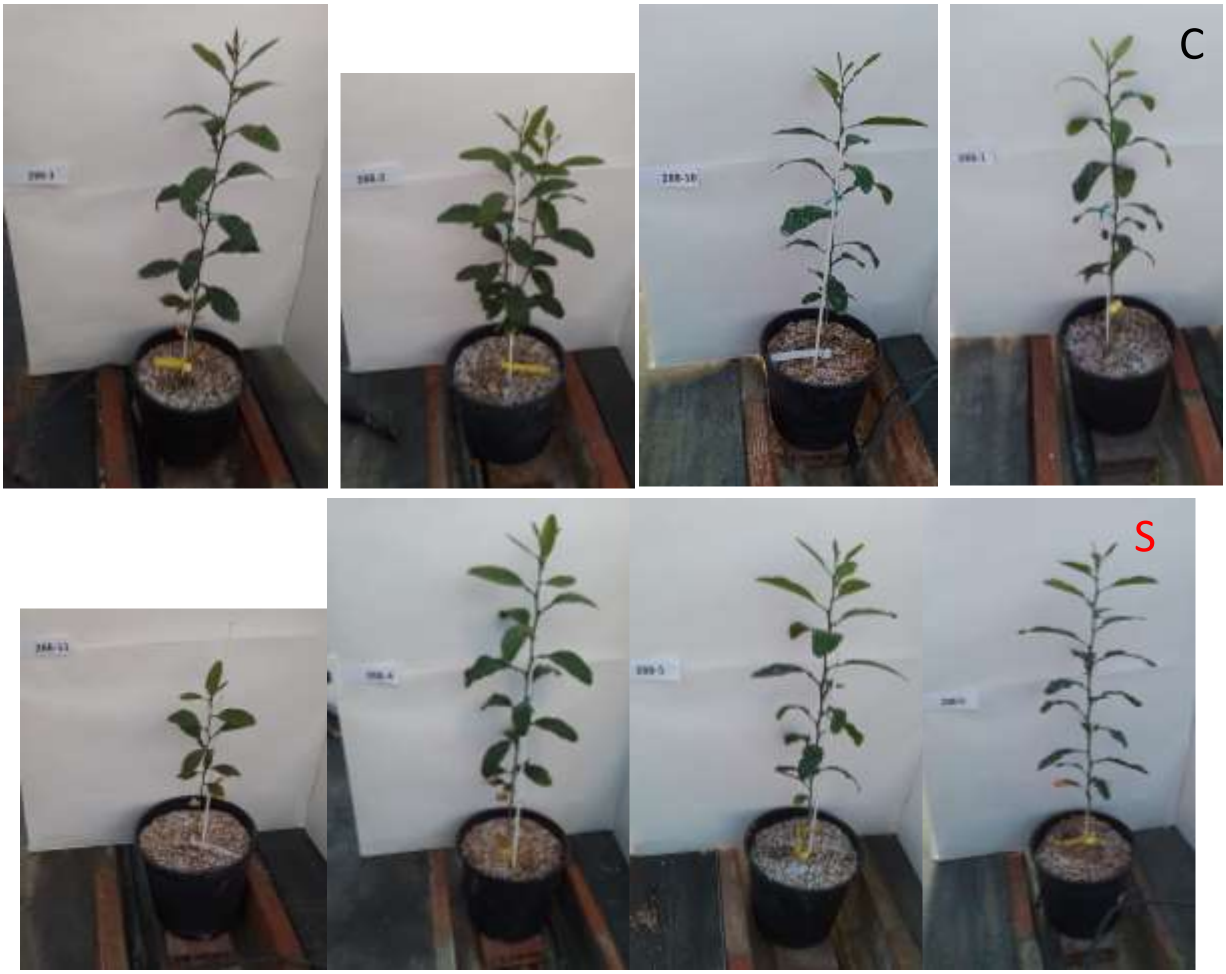

Ra-334 Rangpur lime

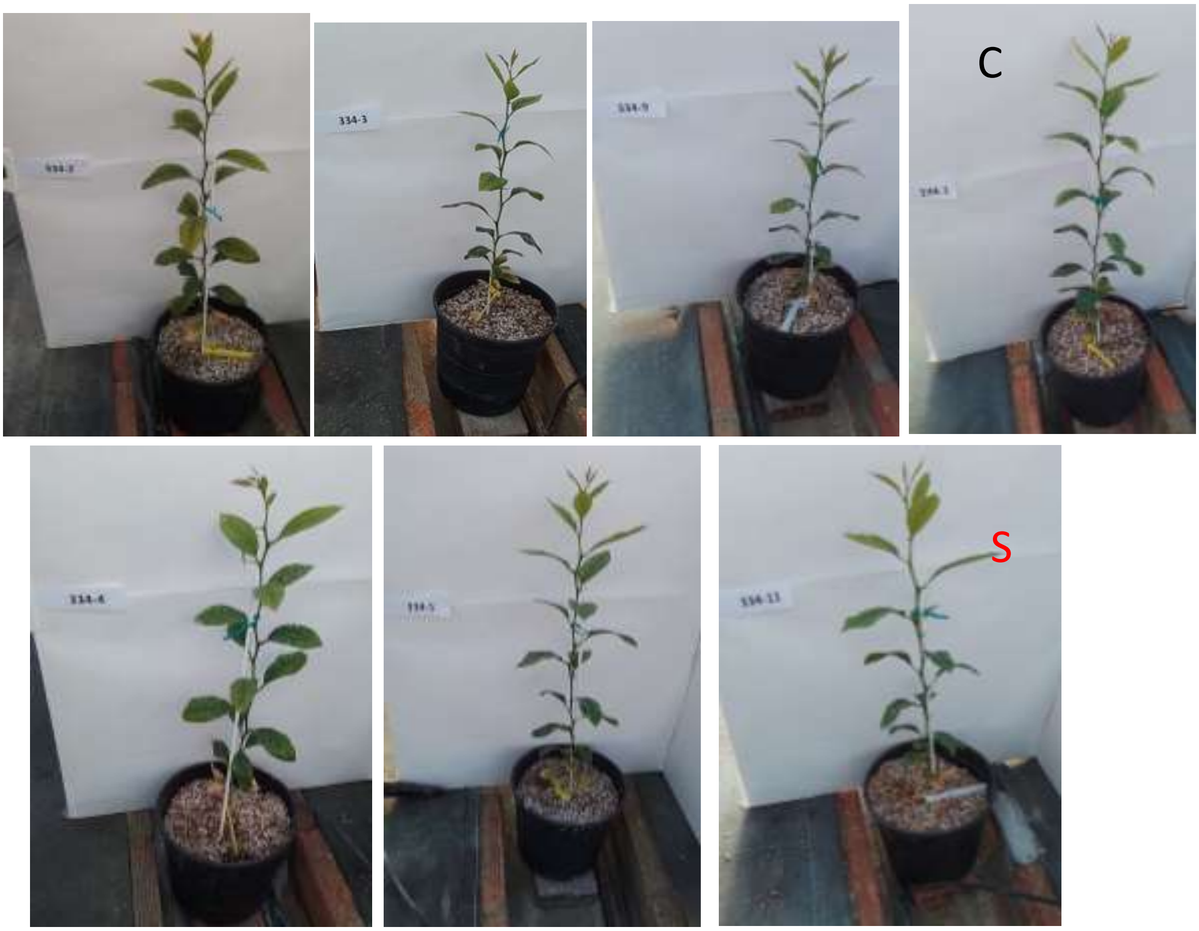

Ru-333 Rough lemon

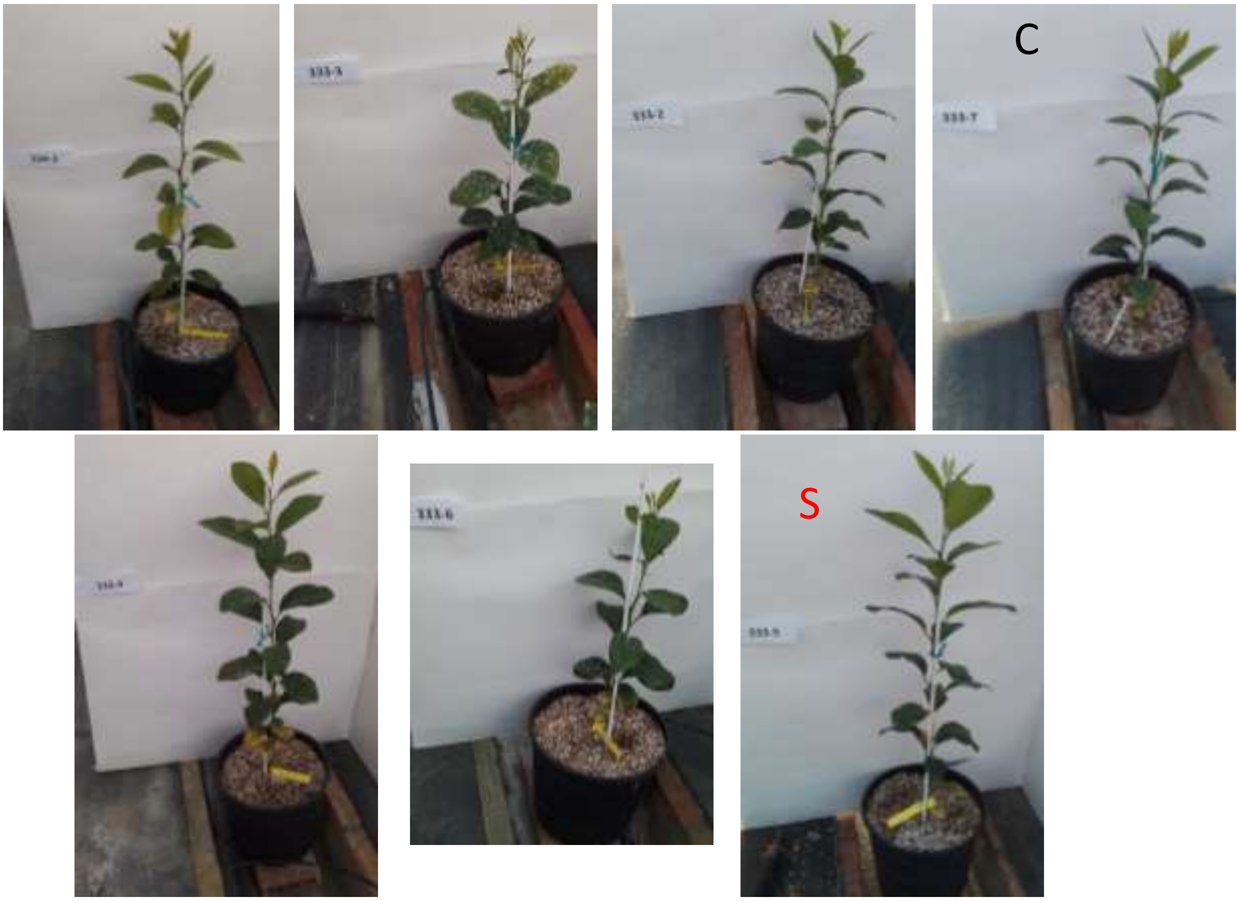

Vo-432 Volkamer lemon

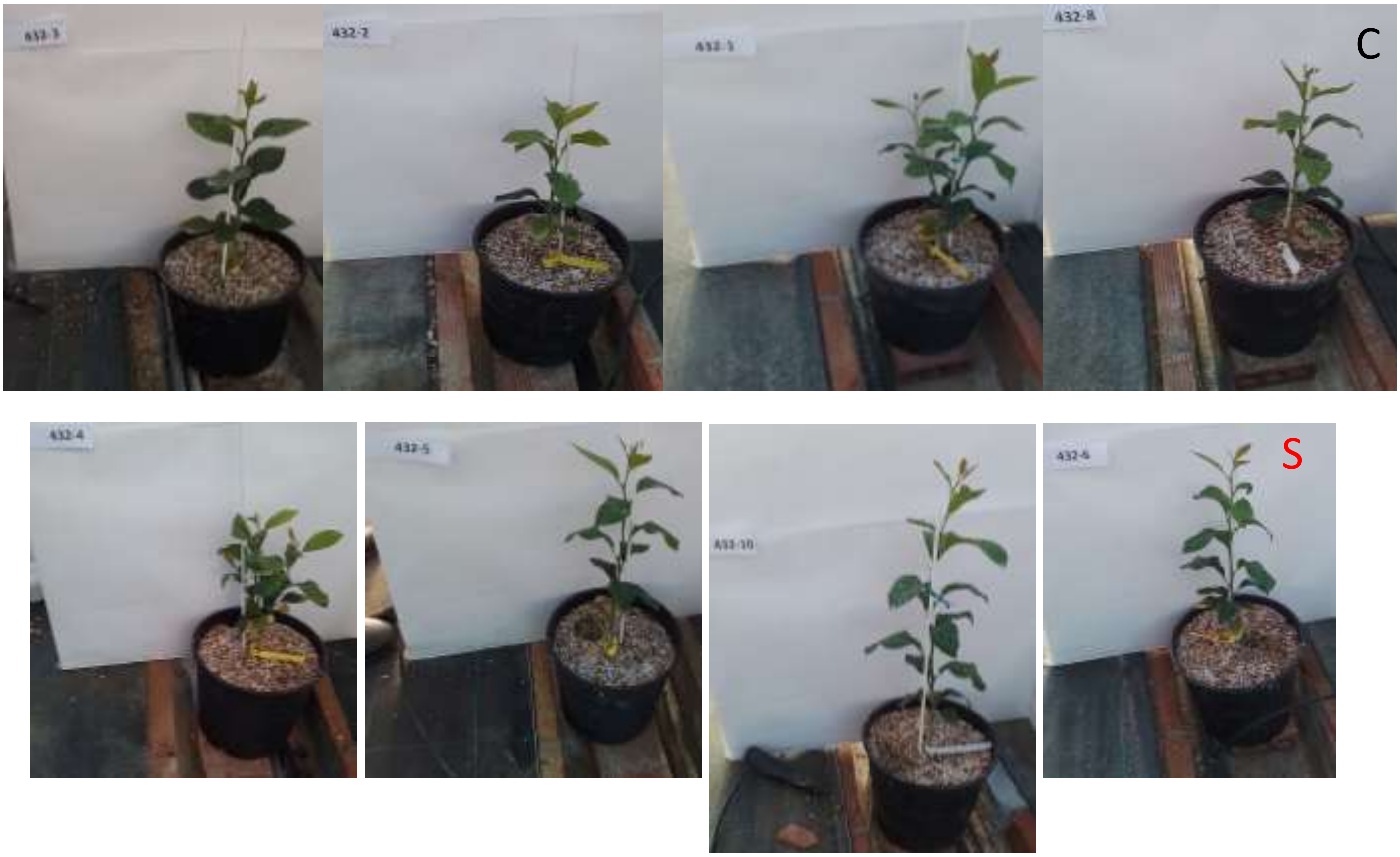

Figure S2. Accession means and standard deviations for root to total plant dry weight ratio (RDW/tPDW). White and blue squares indicate control and salinity conditions, respectively.

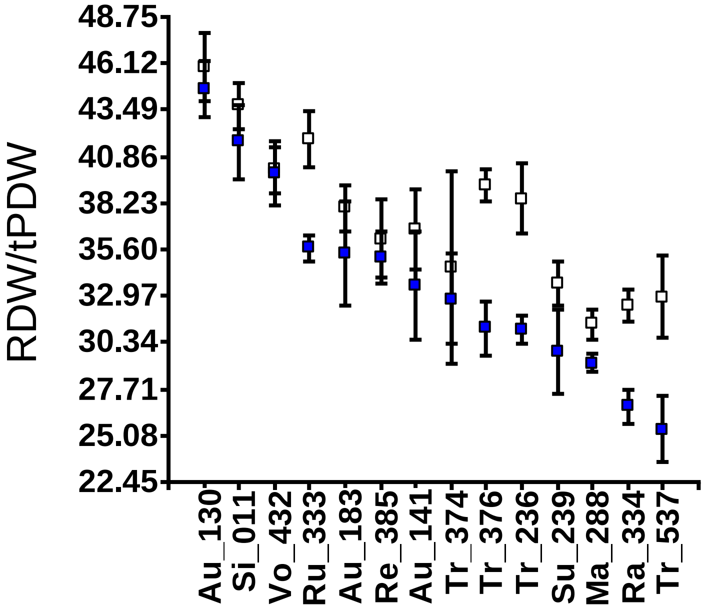

Figure S3. Phenotypic dendrograms of all accessions from the whole collection using their evaluation for vegetative and Cl^- traits under both treatments (A), and each one separately (B, salinity and C, control).

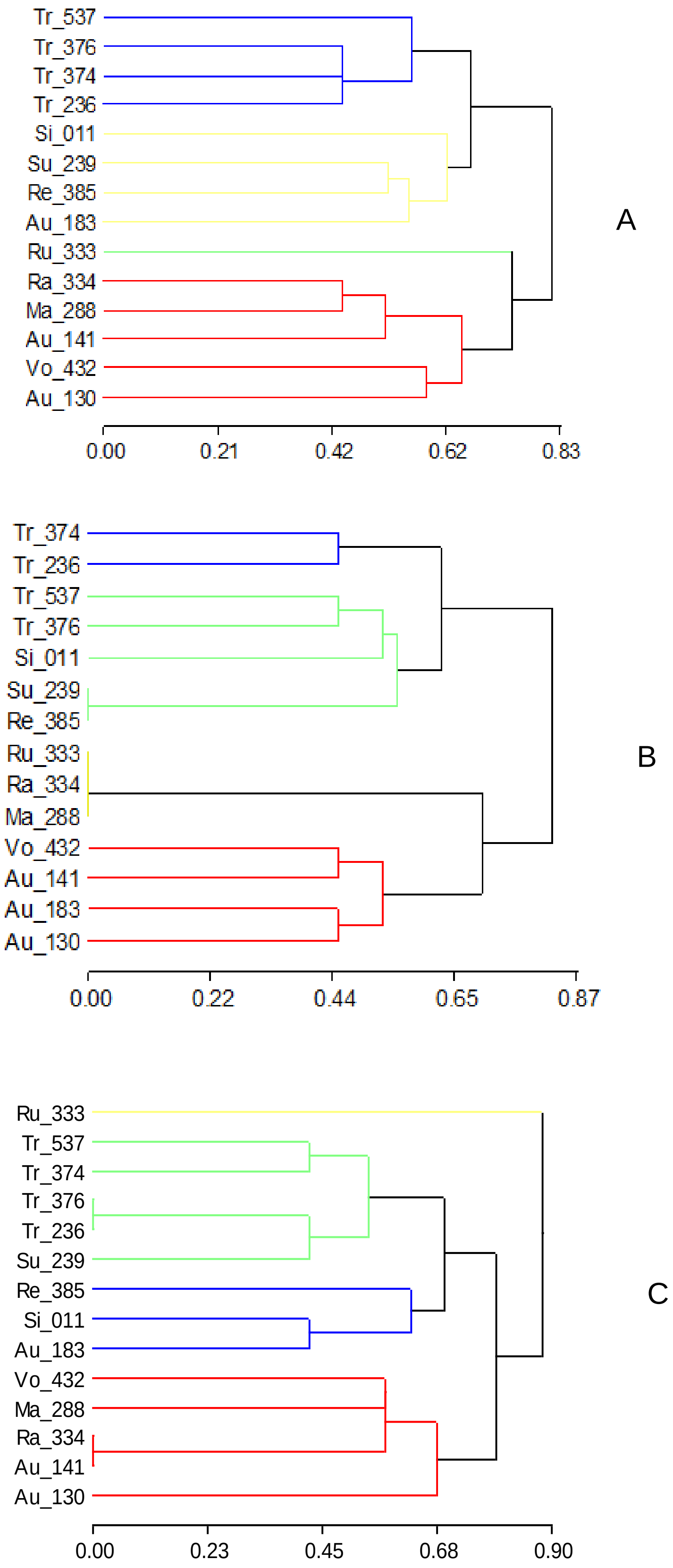

Figure S4. Sequence-characterized amplified region (SCAR) markers (NPF5.9, NPF5.12, MFS, CHX20, ABCG and PIP2.1) and microsatellite CR23 revealed by silver-stained polyacrylamide gel electrophoresis in several plants of each accession ordered by species (codes in Table 1). Arrow at band of PIP2.1 pattern in Tr-537 indicates a sequence difference regarding the other trifoliate accessions.

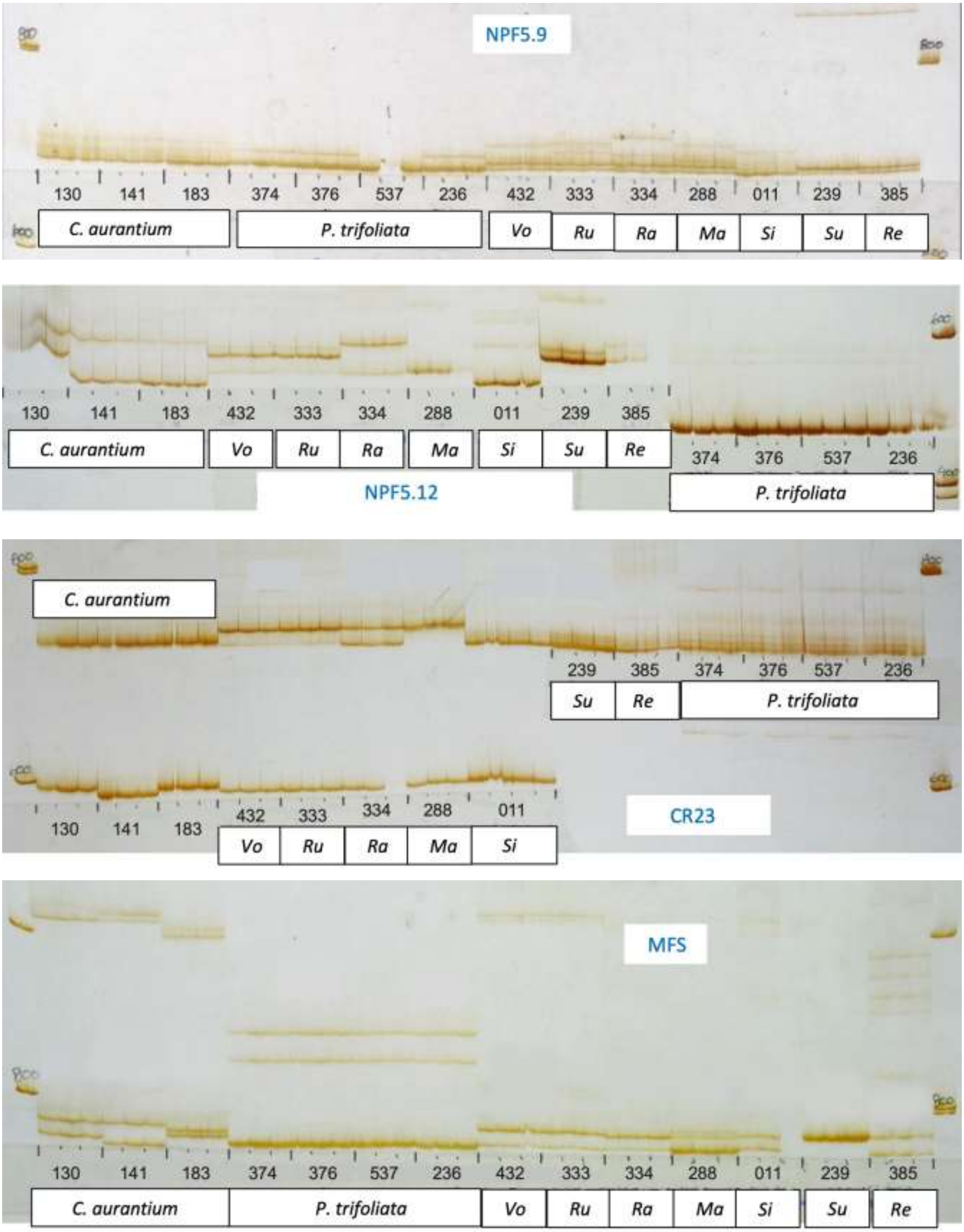

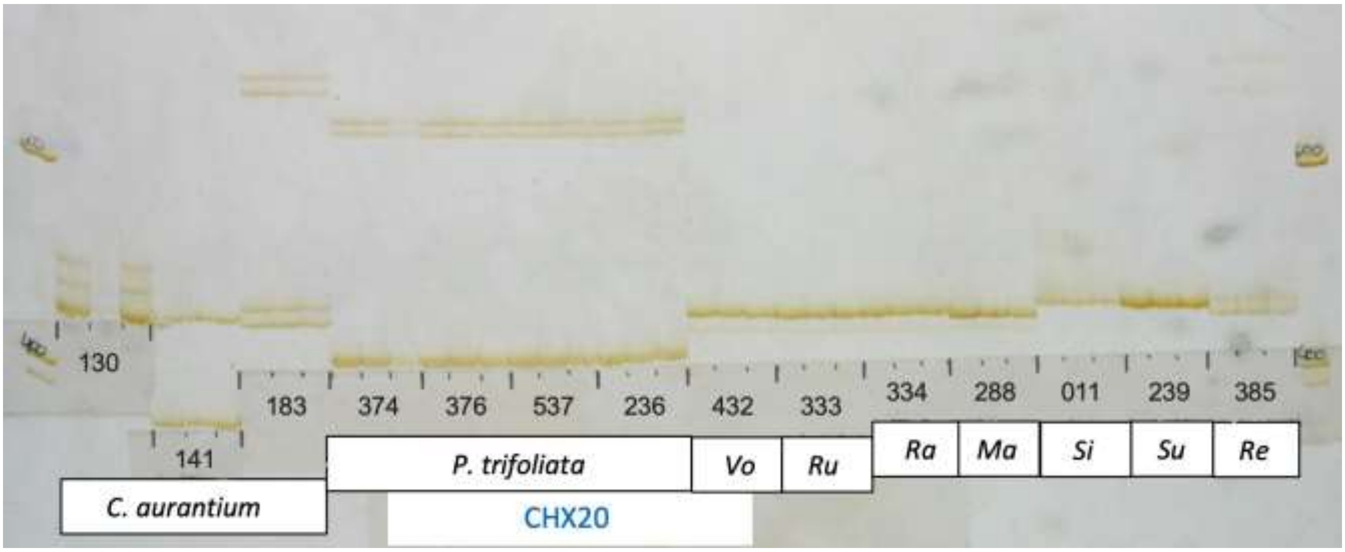
130
141
183
374
376
537
236
432
333
334
288
011
239
385
C. aurantium
P. trifoliata
Vo
Ru
Ra
Ma
Si
Su
Re
CHX20

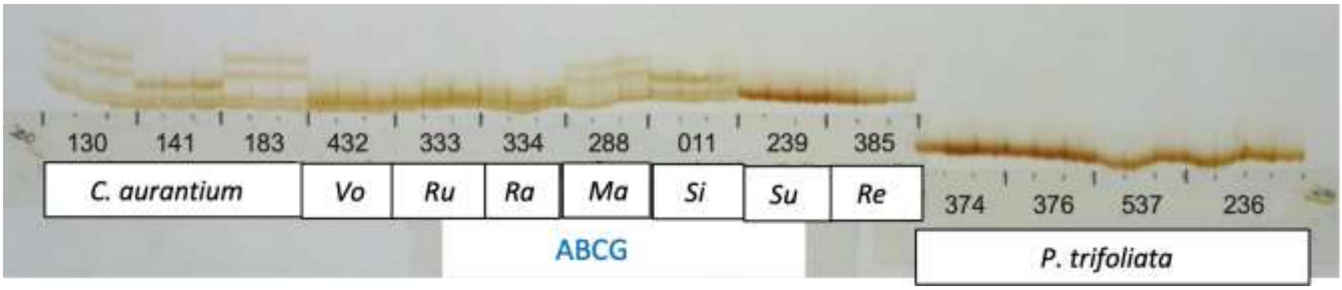
130
141
183
432
333
334
288
011
239
385
374
376
537
236
C. aurantium
Vo
Ru
Ra
Ma
Si
Su
Re
P. trifoliata
ABCG

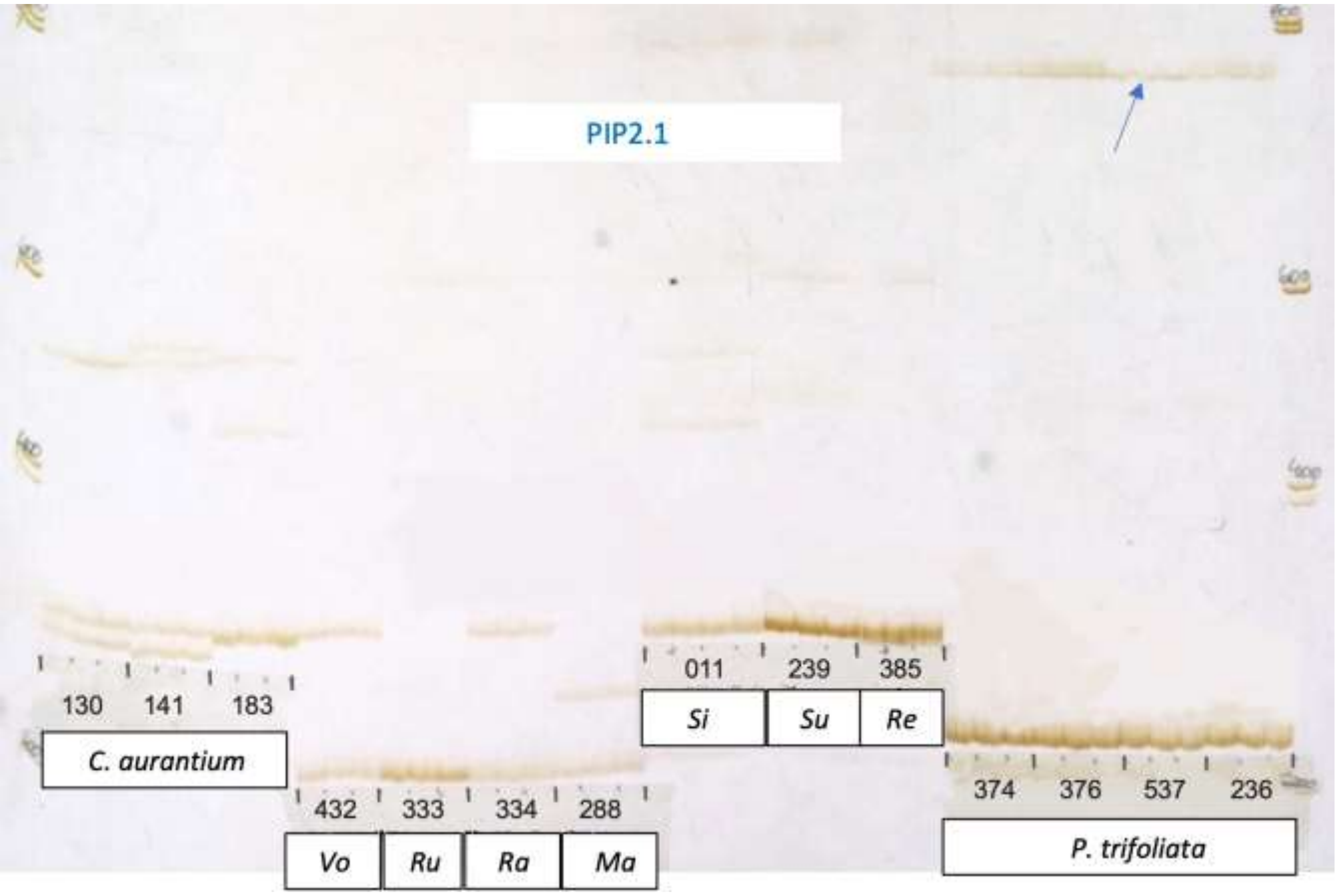
PIP2.1
130
141
183
C. aurantium
432
333
334
288
Vo
Ru
Ra
Ma
011
239
385
Si
Su
Re
374
376
537
236
P. trifoliata

Figure S5. Genotypic dendrograms of accessions from the whole collection using the allele composition at all candidate genes, or at each one separately: NPF5.9, ABCG, PIP2.1, NPF5.12, MFS and CHX20, and at microsatellite CR23.

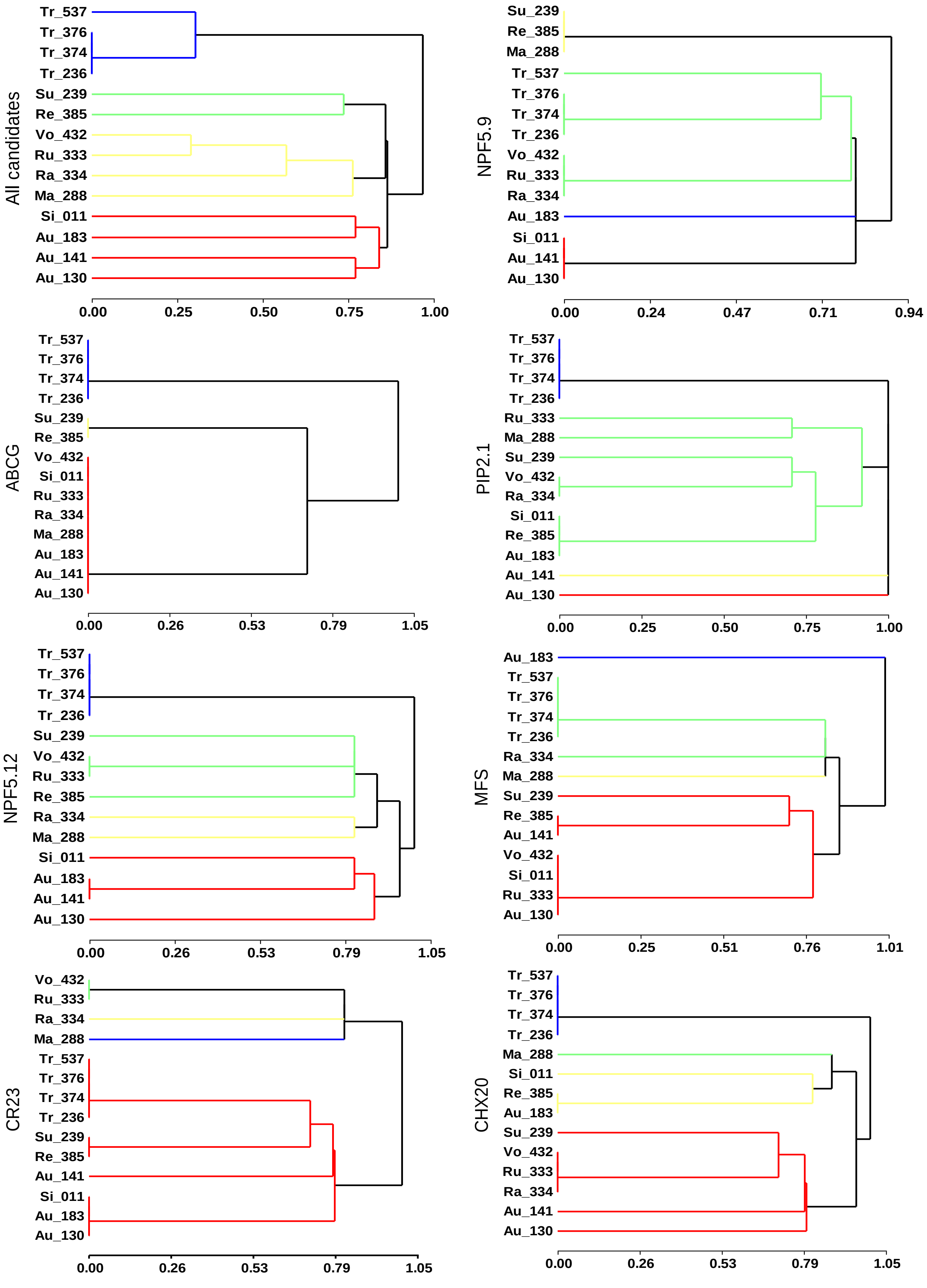

Table S1. Forward and reverse primers for the SCAR targeting candidate gene CHX20 (Ciclev100110060).

1060z1_F	**CACGCCACCTCTACTGTTCC**
1060z1_R	**TCATTCAACGTGCGAGTTTC**

Table S2. Classification of all accessions into groups (1, common mean group; 0, different group) following mean comparisons for vegetative and Cl^- traits.

Trait_Treat_Class	Au_130	Au_141	Au_183	Tr_374	Tr_376	Tr_537	Tr_236	Vo_432	Ru_333	Ra_334	Ma_288	Si_011	Su_239	Re_385
L_Cl_C_A	0	0	0	1	0	1	0	0	0	0	0	0	0	0
L_Cl_C_B	1	1	1	0	1	0	1	1	1	1	1	1	1	1
L_Cl_S_A	0	0	0	1	0	0	1	0	0	0	0	0	0	0
L_Cl_S_B	1	1	1	0	1	1	0	1	1	1	1	1	1	1
R_Cl_C_A	0	0	0	0	0	0	0	1	1	0	0	0	0	1
R_Cl_C_B	1	1	1	1	1	1	1	0	0	1	1	1	1	0
R_Cl_S_A	0	0	0	0	0	1	0	0	0	0	0	0	0	0
R_Cl_S_B	1	1	1	1	1	0	1	1	1	1	1	1	1	1
RCl-LCl/RCl_S_A	1	1	1	1	1	1	0	1	1	1	1	1	1	1
RCl-LCl/RCl_S_B	0	0	0	0	0	0	1	0	0	0	0	0	0	0
dLN_C_A	1	1	1	1	1	1	1	1	1	1	1	1	1	0
dLN_C_B	0	0	0	0	0	0	0	0	0	0	0	0	0	1
SDW_C_A	0	0	0	0	0	0	0	0	1	0	1	0	0	0
SDW_C_B	1	1	0	0	0	0	0	1	0	1	0	0	0	0
SDW_C_C	0	0	1	1	1	1	1	0	0	0	0	1	1	1
SDW_S_A	0	1	0	0	0	0	0	1	1	1	1	0	0	0
SDW_S_B	1	0	1	1	1	1	1	0	0	0	0	1	1	1
RDW_C_A	1	0	0	0	0	0	0	0	1	0	0	0	0	0
RDW_C_B	0	1	1	0	0	0	0	1	0	1	1	1	0	1
RDW_C_C	0	0	0	1	1	1	1	0	0	0	0	0	1	0
RDW_S_A	1	1	1	0	0	0	0	1	1	1	1	0	0	0
RDW_S_B	0	0	0	1	1	1	1	0	0	0	0	1	1	1
tPDW_C_A	0	0	0	0	0	0	0	0	1	0	0	0	0	0
tPDW_C_B	1	1	0	0	0	0	0	1	0	1	1	0	0	0
tPDW_C_C	0	0	1	1	1	1	1	0	0	0	0	1	1	1
tPDW_S_A	0	0	0	0	0	0	0	0	1	1	1	0	0	0
tPDW_S_B	1	1	1	1	1	1	1	1	0	0	0	1	1	1
RDW/tPDW_C_A	1	0	0	0	0	0	0	0	1	0	0	1	0	0
RDW/tPDW_C_B	0	1	1	1	1	1	1	1	0	1	1	0	1	1
RDW/tPDW_S_A	1	0	0	0	0	0	0	1	0	0	0	1	0	0
RDW/tPDW_S_B	0	1	1	1	1	1	1	0	1	1	1	0	1	1
SD_C_A	1	0	0	0	0	0	0	1	1	0	1	0	0	0
SD_C_B	0	1	1	1	1	1	1	0	0	1	0	1	1	1
SD_S_A	1	1	1	0	0	0	0	1	1	1	1	0	0	0
SD_S_B	0	0	0	1	1	1	1	0	0	0	0	1	1	1
dSD_C_A	1	1	1	1	1	0	1	1	1	1	1	1	1	1
dSD_C_B	0	0	0	0	0	1	0	0	0	0	0	0	0	0
LDW_C_A	0	0	0	0	0	0	0	0	1	0	0	0	0	0
LDW_C_B	1	1	1	0	0	0	0	1	0	1	1	1	1	1
LDW_C_C	0	0	0	1	1	1	1	0	0	0	0	0	0	0
LDW_S_A	0	0	0	0	0	0	0	0	1	1	1	0	0	0
LDW_S_B	1	1	1	0	0	0	0	1	0	0	0	0	1	1
LDW_S_C	0	0	0	1	1	1	1	0	0	0	0	1	0	0

Table S3. Classification of accessions into groups (1, common mean group; 0, different group) from the within-species collection following mean comparisons for leaf cation traits.

Trait_Treat_Class	Au_130	Au_141	Au_183	Tr_374	Tr_376	Tr_537	Tr_236	Si_011	Re_385
L_B_C_A	0	0	0	1	1	0	1	0	0
L_B_C_B	1	1	1	0	0	1	0	1	1
L_Ca_C_A	1	1	1	0	0	0	0	1	1
L_Ca_C_B	0	0	0	1	1	1	1	0	0
L_Ca_S_A	1	1	1	0	0	0	0	1	1
L_Ca_S_B	0	0	0	1	1	1	1	0	0
L_K_C_A	1	0	0	0	0	0	0	1	1
L_K_C_B	0	1	1	1	1	1	1	0	0
L_K_S_A	1	0	0	0	0	0	0	1	0
L_K_S_B	0	1	1	1	1	1	1	0	1
L_Mg_C_A	1	0	0	0	0	0	0	0	0
L_Mg_C_B	0	1	0	1	1	1	1	1	1
L_Mg_C_C	0	0	1	0	0	0	0	0	0
L_Mg_S_A	1	0	0	0	0	0	0	0	0
L_Mg_S_B	0	1	1	1	1	1	1	1	1
L_Na_C_A	0	1	0	0	0	0	0	1	0
L_Na_C_B	0	0	0	0	0	0	0	0	1
L_Na_C_C	1	0	1	1	1	1	1	0	0
L_Na_S_A	1	1	0	0	0	1	0	1	1
L_Na_S_B	0	0	1	1	1	0	1	0	0
L_P_C_A	0	0	0	1	1	1	0	0	0
L_P_C_B	1	1	1	0	0	0	1	1	1
L_P_S_A	0	0	0	1	1	1	1	0	0
L_P_S_B	1	1	1	0	0	0	0	1	1
L_S_C_A	0	0	0	1	1	1	1	1	1
L_S_C_B	1	1	1	0	0	0	0	0	0
L_S_S_A	0	0	0	1	1	0	1	0	0
L_S_S_B	1	1	1	0	0	1	0	1	1
L_Cl_C_A	0	0	0	1	0	1	0	0	0
L_Cl_C_B	1	1	1	0	1	0	1	1	1
L_Cl_S_A	0	0	0	1	0	0	1	0	0
L_Cl_S_B	1	1	1	0	1	1	0	1	1
R_Cl_C_A	0	0	0	0	0	0	0	0	1
R_Cl_C_B	1	1	1	1	1	1	1	1	0
R_Cl_S_A	0	0	0	0	0	1	0	0	0
R_Cl_S_B	1	1	1	1	1	0	1	1	1
RCl-LCl/RCl_S_A	1	1	1	1	1	1	0	1	1
RCl-LCl/RCl_S_B	0	0	0	0	0	0	1	0	0
dLN_C_A	1	1	1	1	1	1	1	1	0
dLN_C_B	0	0	0	0	0	0	0	0	1
SDW_C_A	0	0	0	0	0	0	0	0	0
SDW_C_B	1	1	0	0	0	0	0	0	0
SDW_C_C	0	0	1	1	1	1	1	1	1

SDW_S_A	0	1	0	0	0	0	0	0	0
SDW_S_B	1	0	1	1	1	1	1	1	1
RDW_C_A	1	0	0	0	0	0	0	0	0
RDW_C_B	0	1	1	0	0	0	0	1	1
RDW_C_C	0	0	0	1	1	1	1	0	0
RDW_S_A	1	1	1	0	0	0	0	0	0
RDW_S_B	0	0	0	1	1	1	1	1	1
tPDW_C_A	0	0	0	0	0	0	0	0	0
tPDW_C_B	1	1	0	0	0	0	0	0	0
tPDW_C_C	0	0	1	1	1	1	1	1	1
tPDW_S_A	0	0	0	0	0	0	0	0	0
tPDW_S_B	1	1	1	1	1	1	1	1	1
RDW/tPDW_C_A	1	0	0	0	0	0	0	1	0
RDW/tPDW_C_B	0	1	1	1	1	1	1	0	1
RDW/tPDW_S_A	1	0	0	0	0	0	0	1	0
RDW/tPDW_S_B	0	1	1	1	1	1	1	0	1
SD_C_A	1	0	0	0	0	0	0	0	0
SD_C_B	0	1	1	1	1	1	1	1	1
SD_S_A	1	1	1	0	0	0	0	0	0
SD_S_B	0	0	0	1	1	1	1	1	1
dSD_C_A	1	1	1	1	1	0	1	1	1
dSD_C_B	0	0	0	0	0	1	0	0	0
LDW_C_A	0	0	0	0	0	0	0	0	0
LDW_C_B	1	1	1	0	0	0	0	1	1
LDW_C_C	0	0	0	1	1	1	1	0	0
LDW_S_A	0	0	0	0	0	0	0	0	0
LDW_S_B	1	1	1	0	0	0	0	0	1
LDW_S_C	0	0	0	1	1	1	1	1	0

Table S4. Allele composition of all accessions at SCAR markers targeting MFS, NPF5.9, PIP2.1, NPF2.12, ABCG, CHX20 and microsatellite CR23. 1 means presence, 0, absence.

ALLELES	Au_130	Au_141	Au_183	Tr_374	Tr_376	Tr_537	Tr_236	Vo_432	Ru_333	Ra_334	Ma_288	Si_011	Su_239	Re_385
MFS_a	1	1	0	0	0	0	0	1	1	0	0	1	2	1
MFS_b	0	0	1	0	0	0	0	0	0	0	0	0	0	0
MFS_c	0	0	1	0	0	0	0	0	0	0	0	0	0	0
MFS_d	1	0	0	1	1	1	1	1	1	1	1	1	0	0
MFS_e	0	1	0	1	1	1	1	0	0	0	0	0	0	1
MFS_f	0	0	0	0	0	0	0	0	0	1	0	0	0	0
MFS_g	0	0	0	0	0	0	0	0	0	0	1	0	0	0
NPF5.9_a	1	1	0	0	0	0	0	0	0	0	1	1	1	1
NPF5.9_b	1	1	1	1	1	2	1	1	1	1	0	1	0	0
NPF5.9_c	0	0	1	0	0	0	0	0	0	0	0	0	0	0
NPF5.9_d	0	0	0	1	1	0	1	0	0	0	0	0	0	0
NPF5.9_e	0	0	0	0	0	0	0	1	1	1	1	0	1	1
PIP2.1_a	1	0	0	0	0	0	0	0	0	0	0	0	0	0
PIP2.1_b	0	1	0	0	0	0	0	0	0	0	0	0	0	0
PIP2.1_c	0	0	1	0	0	0	0	1	0	1	0	1	2	1
PIP2.1_d	0	0	1	0	0	0	0	0	0	0	0	1	0	1
PIP2.1_e	1	0	0	0	0	0	0	0	0	0	0	0	0	0
PIP2.1_f	0	1	0	0	0	0	0	0	0	0	0	0	0	0
PIP2.1_g	0	0	0	1	1	1	1	0	0	0	0	0	0	0
PIP2.1_h	0	0	0	1	1	1	1	0	0	0	0	0	0	0
PIP2.1_i	0	0	0	0	0	0	0	1	2	1	1	0	0	0
PIP2.1_j	0	0	0	0	0	0	0	0	0	0	1	0	0	0
NPF5.12_a	1	1	1	0	0	0	0	0	0	1	0	0	0	1
NPF5.12_b	1	0	0	0	0	0	0	0	0	0	0	0	0	0
NPF5.12_c	0	1	1	0	0	0	0	0	0	0	0	1	0	0
NPF5.12_d	0	0	0	1	1	1	1	0	0	0	0	0	0	0
NPF5.12_e	0	0	0	1	1	1	1	0	0	0	0	0	0	0
NPF5.12_f	0	0	0	0	0	0	0	1	1	0	0	0	1	1
NPF5.12_g	0	0	0	0	0	0	0	1	1	1	1	0	0	0
NPF5.12_h	0	0	0	0	0	0	0	0	0	0	1	0	0	0
NPF5.12_i	0	0	0	0	0	0	0	0	0	0	0	0	1	0
NPF5.12_j	0	0	0	0	0	0	0	0	0	0	0	1	0	0
ABCG_a	1	1	1	0	0	0	0	1	1	1	1	1	0	0
ABCG_b	1	1	1	0	0	0	0	1	1	1	1	1	2	2
ABCG_c	0	0	0	2	2	2	2	0	0	0	0	0	0	0
CHX20_a	1	1	0	0	0	0	0	1	1	1	0	0	2	0
CHX20_b	0	0	1	0	0	0	0	0	0	0	0	1	0	1
CHX20_c	1	0	1	0	0	0	0	0	0	0	1	0	0	1
CHX20_d	0	1	0	0	0	0	0	0	0	0	0	0	0	0
CHX20_e	0	0	0	1	1	1	1	0	0	0	0	0	0	0
CHX20_f	0	0	0	1	1	1	1	0	0	0	0	0	0	0
CHX20_g	0	0	0	0	0	0	0	1	1	1	1	0	0	0
CHX20_h	0	0	0	0	0	0	0	0	0	0	0	1	0	0
CR23_a	1	1	1	1	1	1	1	0	0	0	0	1	2	2
CR23_b	1	0	1	0	0	0	0	0	0	0	0	1	0	0
CR23_c	0	1	0	0	0	0	0	0	0	0	0	0	0	0
CR23_d	0	0	0	1	1	1	1	0	0	0	0	0	0	0
CR23_e	0	0	0	0	0	0	0	1	1	0	0	0	0	0
CR23_f	0	0	0	0	0	0	0	1	1	1	1	0	0	0
CR23_g	0	0	0	0	0	0	0	0	0	1	0	0	0	0
CR23_h	0	0	0	0	0	0	0	0	0	0	1	0	0	0
